# Plaque Characteristics in Coronary Artery Disease Patients with Impaired Glucose Tolerance

**DOI:** 10.1371/journal.pone.0167645

**Published:** 2016-12-09

**Authors:** Keishi Suzuki, Hitoshi Takano, Yoshiaki Kubota, Keisuke Inui, Shunichi Nakamura, Yukichi Tokita, Koji Kato, Kuniya Asai, Wataru Shimizu

**Affiliations:** Department of Cardiovascular Medicine, Nippon Medical School, Tokyo, Japan; University of Louisville, UNITED STATES

## Abstract

**Background:**

Impaired glucose tolerance (IGT) patients are known to have a high risk of cardiovascular events and their prognosis has been reported to be poor. The present study aimed to compare coronary plaque characteristics among coronary artery disease (CAD) patients with normal glucose tolerance (NGT), those with IGT, and those with diabetes mellitus (DM) by using optical coherence tomography (OCT).

**Methods:**

The present study included 101 coronary artery disease patients (mean age, 67.9 ± 10.4 years; 82.4% male). OCT was performed for target and non-target vessels during percutaneous coronary intervention. The patients were divided into the following 3 groups: the NGT, IGT, and DM groups.

**Results:**

A total of 136 non-target residual plaques were found in 101 patients (27, 30, and 44 in the NGT, IGT, and DM groups, respectively). The size of the lipid core expressed as the mean angle of the lipid arc was significantly greater in the IGT and DM groups than in the NGT group (163.0 ± 58.7°, 170.1 ± 59.3°, and 130.9 ± 37.7°, respectively, P < 0.05). The fibrous cap covering the lipid core was significantly thinner in the IGT group than in the NGT group (77.0 ± 23.4 μm vs. 105.6 ± 47.0 μm, P = 0.040).

**Conclusion:**

The coronary plaques in CAD patients are more vulnerable when having IGT compared to those with NGT, and similar to those with DM. This finding may explain the high risk of cardiovascular events in CAD patients with IGT.

## Background

In addition to diabetes mellitus (DM), which is established as one of the most significant risk factors of coronary artery disease (CAD), prediabetic conditions such as impaired glucose tolerance (IGT) have been also reported to increase the risk of cardiovascular events and affect mortality rates [1, 2]. Previous studies have reported that severe atherosclerotic lesions are frequently observed in prediabetic patients suggesting the atherosclerotic formation starts at a very early stage [3–8]. As well as the severity and extent of atherosclerotic lesions, the morphology of atherosclerotic plaque is also considered to be one of the important factors responsible for the development of cardiovascular events [9, 10]. Vulnerable coronary plaques characterized by thin-cap fibroatheroma (TCFA), large lipid core, and macrophages infiltration are closely associated with the onset of acute coronary syndrome (ACS) [11–13]. Optical coherence tomography (OCT) is a high-resolution intravascular imaging modality that enables detailed assessment of coronary plaque morphology, which is considered to be superior to any other modalities currently available [14–16]. A previous study reported that DM patients have a larger lipid core in their coronary plaques and poorly controlled DM patients had more TCFA on OCT, which may be explaining the high risk of cardiac events [17]. The phenomenon similar to that observed in DM patients in their study can be also occurring even in CAD patients with IGT. Thus, the morphological assessment of atherosclerotic plaque by OCT may also be helpful to understand the mechanism of development of cardiovascular events in CAD patients with IGT.

In the present study, we aimed to evaluate the vulnerability of coronary plaques in CAD patients with IGT using OCT and compare it with those with normal glucose tolerance (NGT) or DM. The data will clarify whether the coronary plaques in CAD patients with IGT are more vulnerable than those in the patients with NGT and how different they are from those in the patients with DM.

## Research Design and Methods

### Patient population

**Fig 1:**
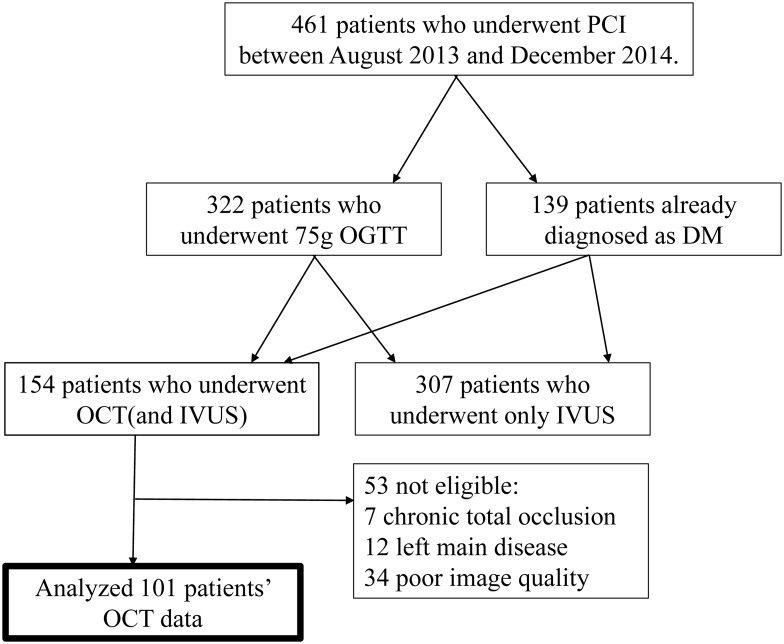
Flow chart of patients we analyzed in the present study. PCI: percutaneous coronary intervention, OGTT: oral glucose tolerance test, DM: diabetes mellitus, OCT: optical coherence tomography, and IVUS: intravascular ultra sound.

There were 461 consecutive CAD patients who received PCI and were registered in our institutional PCI registry between August 2013 and December 2014 (Fig 1). Indication of PCI was determined when the patients have typical effort angina or positive stress-test findings (either ECG, nuclear scan, or stress echocardiogram), and suitable coronary stenotic lesion(s) for PCI. In this registry, the content of PCI procedure including the findings of intravascular imaging and clinical data including the result of 75-g oral glucose tolerance test (OGTT) were recorded. Thus all patients underwent 75-g OGTT except those already diagnosed as DM (Fig 1). 154 patients received OCT of the target and non-target vessels during percutaneous coronary intervention (PCI) by the intention of the operator (Fig 1). Patients with chronic total occlusion (n = 7), left main disease (n = 12), and poor image quality (n = 34) were excluded; therefore, 101 patients were included in the final analysis (Fig 1). Non-culprit lesions were defined as plaques angiographically confirmed but not treated during the session of PCI according to the results of stress test or ECG changes during the spontaneous ischemic attacks. Blood samples were obtained from the antecubital vein in the fasting state and before each OCT procedure. The patients were divided into 3 groups according to the results of a 75-g oral glucose tolerance test (OGTT). Glucose metabolism was assessed according to the American Diabetes Association (ADA) and World Health Organization (WHO) criteria [18].

NGT was defined as a fasting plasma glucose (FPG) level <110 mg/dL and 2-h plasma glucose (PG) level <140 mg/dL during an OGTT. IGT was defined as a 2-h PG level ≥140 mg/dL but <200 mg/dL during an OGTT. DM was defined as a history of DM, hemoglobin A1c (HbA1c) value ≥6.5%, and FPG level ≥126 mg/dL or 2-h PG level ≥200 mg/dL during an OGTT[18]. Height and weight were measured at the time of discharge, and the body mass index (BMI; kg/m^2^) was calculated as an index of obesity. Hypertension was defined as a systolic blood pressure ≥140 mmHg, a diastolic blood pressure ≥90 mmHg, or the current use of antihypertensives. Dyslipidemia was defined as a total cholesterol (TC) level ≥220 mg/dL, low-density lipoprotein-cholesterol (LDL-C) level ≥140 mg/dL, high-density lipoprotein-cholesterol (HDL-C) level <40 mg/dL, or the current use of lipid-lowering agents. Chronic kidney disease was defined as a glomerular filtration rate (GFR) ≤60 mL/min/1.73 m^2^ or proteinuria. The GFR was estimated using the simplified prediction equation derived from the Modification of Diet in a Renal Disease study [19].

### OCT imaging and analysis

OCT images were obtained using frequency-domain OCT (C7-XRTM; LightLab Imaging). With the help of a 6 or 7-Fr guiding catheter, images were obtained using a continuous flush of contrast media or low molecular weight dextran at a rate of 4 mL/s from the guiding catheter, and the OCT wire was pulled back at a rate of 10 mm/s. Images were recorded at 100 frames/s, displayed with a color look-up table, and digitally archived.

We characterized the non-culprit lesions in CAD patients with DM and IGT using OCT imaging and compared them with those in the NGT patients. Each plaque was separated at least 5 mm from the edge of an implanted stent. The plaques were classified as fibrous (homogeneous with a highly backscattered region) or lipid (low signal region with a diffuse border). A thin-cap fibroatheroma (TCFA) was defined as the thinnest fibrous cap with a thickness ≤65 μm in a lipid-rich plaque on cross-sectional imaging. Macrophage infiltration was defined as signal-rich, distinct or confluent punctuate regions that exceed the intensity of background speckle noise [20–22]. Plaque disruption was defined as fibrous cap discontinuity with clear cavity formation inside the plaque [23]. Microchannel structures were defined as signal-poor voids that are sharply delineated in multiple contiguous frames [22]. Calcification was defined as well delineated and low backscattered heterogeneous regions [22]. A thrombus was defined as a well-delineated mass with a high signal attached to the luminal surface or floating within the lumen [22, 23]. OCT images were analyzed by 2 independent observers. The inter-observer reliability between the 2 observers measured by the Pearson coefficient was r = 0.97 (S1 Fig). The intra-observer reproducibility determined by the Pearson coefficient was r = 0.95 (S2 Fig).

The Medical Ethics Committee at Nippon Medical School Hospital approved the study protocol, and written informed consent was obtained from all the patients before the catheterization procedure.

### Statistical analysis

Categorical variables were presented as frequencies, and these were compared using the Pearson chi-square test. Continuous variables are presented as means ± standard deviations, and these were compared using ANOVA. The relationships between the OCT findings, and lipid profiles or HbA1c levels were assessed using Pearson's correlation. All statistical analyses were performed using SPSS software package ver. 20.0 (IBM Corp., Armonk, NY, USA). A p-value <0.05 was considered statistically significant.

## Results

### Baseline characteristics

The baseline characteristics of the patients are presented in Table 1. The NGT group included 27 patients (26.7%), the IGT group included 30 patients (29.7%), and the DM group included 44 patients (43.6%, 24 patients were known to have DM). The HbA1c level was significantly higher in the DM group (7.1 ± 0.8%) than in the IGT (5.9 ± 0.3%, P < 0.01) and NGT group (5.6 ± 0.5%, P < 0.01). There were no significant differences in the LDL-C level, HDL-C level, and statin use among the 3 groups.

**Table 1:** Clinical characteristics of the study population.

Characteristic	NGT (n = 27)	IGT (n = 30)	DM (n = 44)	P-value
Age, years	66.1 ± 9.8	69.3 ± 12.2	67.2 ± 9.2	0.485
Male sex, n (%)	21 (81.5%)	23 (76.7%)	37 (84.1%)	0.73
BMI, kg/m^2^	23.3 ± 2.7	25.0 ± 4.1	24.6 ± 4.5	0.277
Hypertension, n (%)	25 (92.6%)	27 (90%)	40 (90.9%)	0.943
Dyslipidemia, n (%)	18 (66.7%)	19 (63.3%)	27 (61.4%)	0.906
Smoking, n (%)	10 (39.1%)	17 (56.7%)	28 (63.6%)	0.161
ACS, n (%)	7 (25.9%)	8 (26.7%)	8 (18.2%)	0.676
Prior MI, n (%)	12 (44.4%)	8 (26.7%)	15 (34.1%)	0.459
HbA1c, %	5.6 ± 0.5	5.9 ± 0.3	7.1 ± 0.8	<0.001
FPG, mg/dL	90.6 ± 7.6	96.2 ± 13.3	106.7 ± 17.9	0.003
LDL-C, mg/dL	101.2 ± 32.0	96.7 ± 23.7	93.1 ± 26.9	0.489
HDL-C, mg/dL	51.3 ± 13.6	51.1 ± 17.6	47.1 ± 12.8	0.383
Statin use, n (%)	23 (88.5%)	23 (76.7%)	33 (75%)	0.389

The data are presented as mean ± standard deviation or actual number (percentage)

NGT, normal glucose tolerance; IGT, impaired glucose tolerance; DM, diabetes mellitus; BMI, body mass index; ACS, acute coronary syndrome; MI, myocardial infarction; FPG, fasting plasma glucose; LDL-C, low-density lipoprotein cholesterol; HDL-C, high-density lipoprotein cholesterol.

### OCT findings

The OCT plaque characteristics are presented in Table 2. Of the 136 non-target residual plaques, 72 were found to contain a lipid core on OCT (16, 28, and 28 in the NGT, IGT, and DM groups, respectively). The percentage of lipid-rich plaques was similar among the NGT, IGT and DM groups. Additionally, there were no differences in the percentages of TCFA, macrophage infiltration, plaque disruption, microchannels, calcification, and thrombi among the groups (Table 2).

**Table 2:** Clinical characteristics of the study population on optical coherence tomography.

Characteristic	NGT (n = 27)	IGT (n = 30)	DM (n = 44)	P-value
Total plaques observed by OCT, n	33	44	59	
Lipid-rich plaques, n	16	28	28	
Patient with lipid-rich plaques, n (%)	12 (44.4)	20 (66.6)	18 (40.9)	0.266
Lipid-rich plaques/total plaques, %	48.5	63.6	47.5	0.227
TCFA, n (%)	5 (15.2)	9 (20.5)	11 (18.6)	0.839
Macrophage infiltration, n (%)	0 (0)	4 (9.1)	6 (10.2)	0.177
Disruption, n (%)	2 (6.1)	4 (9.1)	4 (6.8)	0.862
Microchannels, n (%)	10 (30.3)	8 (18.2)	11 (18.6)	0.356
Calcification, n (%)	21 (63.6)	22 (50)	31 (52.5)	0.464
Thrombus, n (%)	4(12.1)	4 (9.1)	6 (10.2)	0.912

Data are expressed as actual number (% of total plaques observed by OCT) otherwise specified.

**Fig 2:**
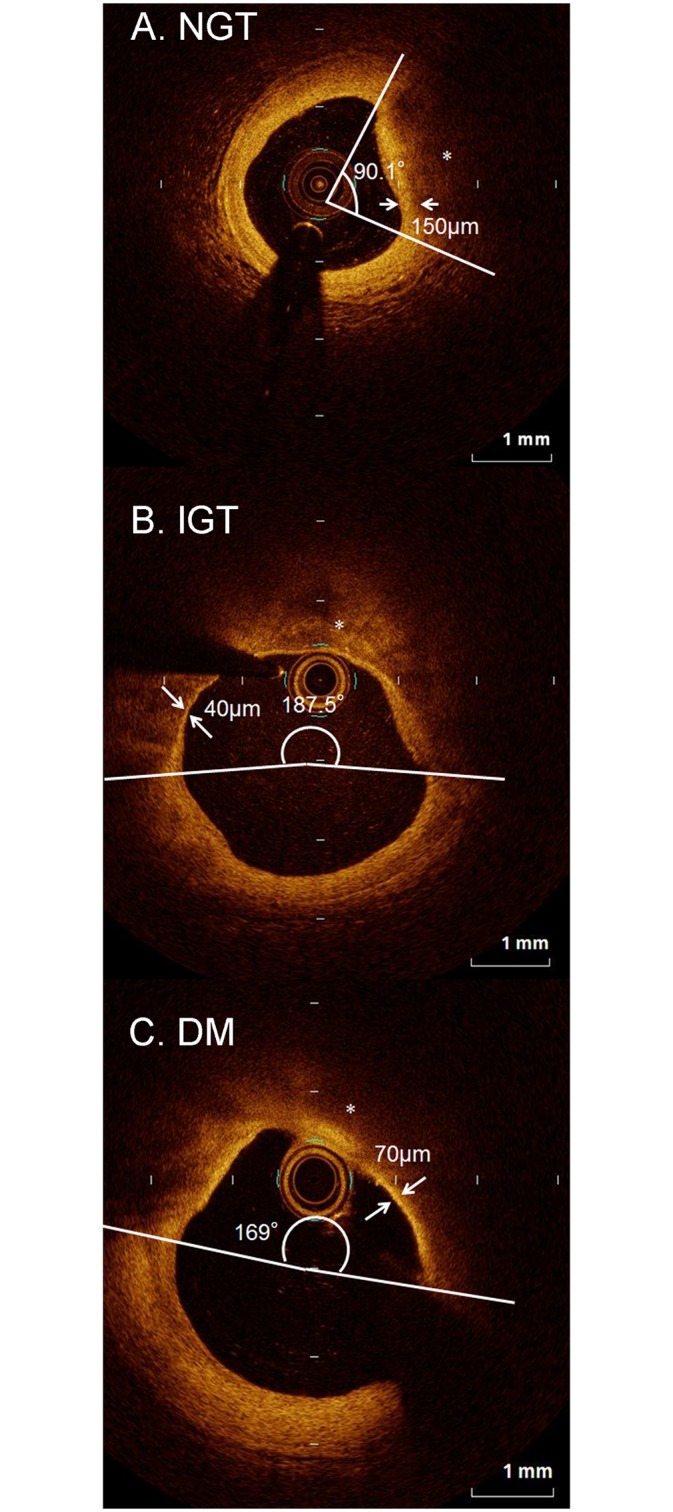
Representative cross-sectional OCT images for each of the 3 groups, NGT, IGT and DM (A, B, and C, respectively). We measured the thickness of the thinnest part (arrows) of the fibrous cap identified as a signal-rich homogenous region overlying a lipid core (*), which is characterized by a signal-poor region.

**Fig 3:**
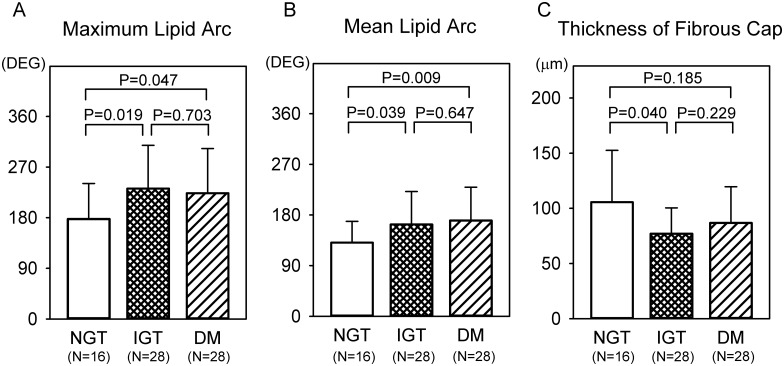
Comparisons of OCT findings of coronary plaques containing lipid core between NGT, IGT, and DM groups. Maximum and mean lipid arc (panels A and B) and thickness of fibrous cap (panel C). Data are expressed as mean ± SD.

**Fig 4:**
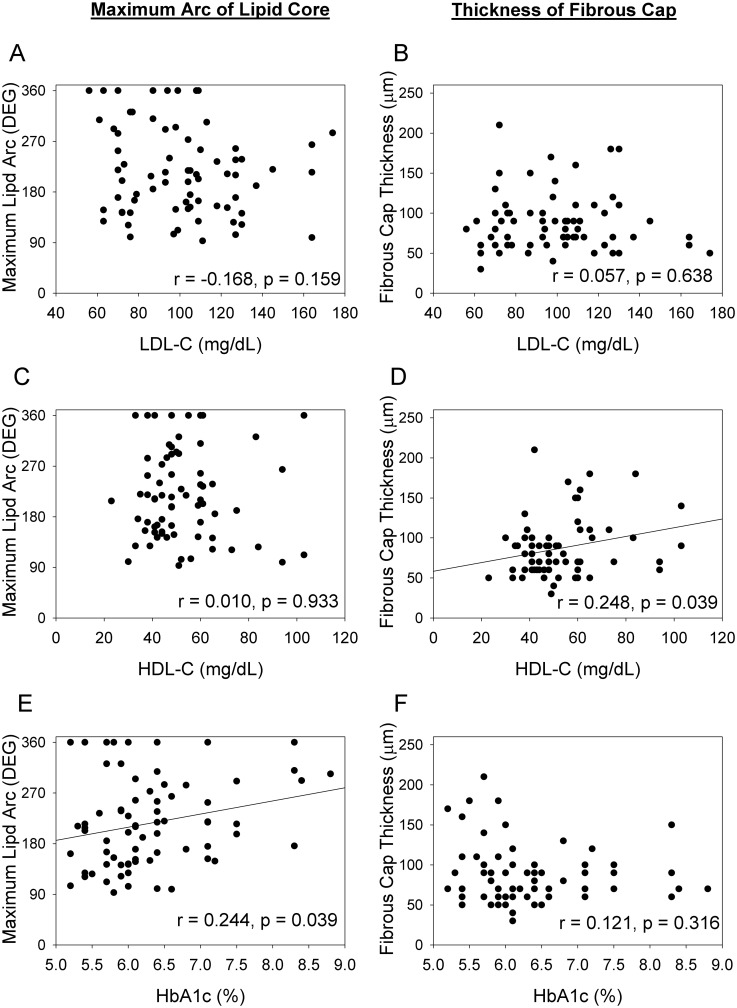
Relationships between OCT findings (maximum lipid arc and fibrous cap thickness) and LDL-C (panels A and B), HDL-C (panels C and D) and HbA1c (panels E and F). Each panel illustrates individual values with closed circles. In the panels D and E, the linear regression lines are expressed. Fibrous cap thickness is positively and linearly correlated with HDL-C (panel D, y = 23.4x+69.2, r = 0.248 and p = 0.039). Maximum lipid arc was positively and linearly correlated with HbA1c (panel E, y = 0.544x+58.3, r = 0.244, p = 0.039). In other panels, only r and p values of linear regression analysis were expressed because correlation was not statistically significant.

Representative OCT images in each group are shown in Fig 2. A comparison of the quantitative OCT findings of the lipid plaques between the NGT, IGT, and DM groups is presented in Fig 3. The plaques had a significantly wider maximum lipid arc and mean lipid arc in the IGT and DM groups than in the NGT group (max lipid arc: 231.8° vs. 177.6°, P = 0.019 and 223.6° vs. 177.6°, P = 0.047; mean lipid arc: 163.0° vs. 130.9°, P = 0.039 and 170.1° vs. 130.9°, P = 0.009, respectively). There were no significant differences in these lipid plaque parameters between the IGT and DM groups. The fibrous cap thickness was lower in the IGT group than in the NGT group (77.0 μm vs. 105.6 μm, P = 0.040) (Fig 3). The relationships between the OCT findings, and lipid profiles or HbA1c level are shown in Fig 4. Max lipid arc positively correlated with HbA1c (y = 23.4x+69.2, r = 0.244, P = 0.039). Fibrous cap thickness positively correlated with HDL-C (y = 0.544x + 58.3, r = 0.248, P = 0.039).

## Discussion

The present study aimed to identify the coronary plaque characterization of CAD patients with IGT defined by the result of a 75 g OGTT. There were no significant differences in the percentage of coronary plaques or TCFAs between NTG, IGT and DM patients. However, a significant difference was observed in the quality of the plaque containing a lipid core. The lipid core in the coronary plaque of CAD patients with IGT was significantly larger and the fibrous cap covering the core was significantly thinner than those of NGT patients and equivalent to those of DM patients.

An increase in the size of the lipid plaque and thinning of the fibrous cap can cause ACS through an increase in atheroma vulnerability. OCT is an imaging modality characterized by clearly identifying plaque rupture, fibrous cap erosion, an intracoronary thrombus, a lipid plaque arc, and TCFA in vivo. OCT has been established as the modality for the detailed plaque morphological analysis, superior to other conventional imaging techniques such as intravascular ultra sound [24]. In a previous study, coronary tissue exhibited a larger number of lipid-rich atheromas, macrophage infiltrations, and thrombus among DM patients than among NGT patients, and patients with HbA_1C_ ≥8% had a wide lipid arc, a long lipid length, thin fibrous caps, and high prevalence of TCFA and macrophage infiltration, which coincided with the typical pathological features of vulnerable plaques[17]. Kurihara et al. demonstrated that coronary atherosclerosis and plaque vulnerability assessed using angioscopy were more advanced in prediabetic patients than in nondiabetic patients[25]. A recent study showed that glucose fluctuations impact not only atherosclerosis, but also the formation of lipid-rich plaques and thinning of the fibrous cap in CAD patients [26]. In the present study, we performed 75g OGTT in all the patients who had not been diagnosed as DM to specify the patients with IGT and demonstrated that plaque vulnerability was more advanced in CAD patients with IGT than those with NGT using OCT.

The mechanisms of the development of vulnerable plaques in hyperglycemic status are not fully understood. Physiological studies have reported that hyperglycemia, excess free fatty acid, and insulin resistance in diabetes can cause metabolic disarray within endothelial cells, and the activation of these systems impairs endothelial function, augments vasoconstriction, increases inflammation, and promotes thrombosis[27]. These conditions may also affect vasoconstriction and inflammation, and thereby promote coronary atherosclerosis even in the prediabetic phase. Several in vitro investigations have reported that the development of oxidative stress and vascular injury are more strongly associated with glucose variability than exposure to a constant high glucose level [28, 29]. Schisano et al. reported that more detrimental conditions in terms of oxidative stress and DNA damage in endothelial cells were noted with prolonged exposure to oscillating glucose levels than exposure to constant high glucose levels, which were associated with hyperactivation of p53, in vitro[30]. These findings suggest that IGT could have an ominous impact on the function of endothelial cells to promote atherosclerosis, leading to an increase in plaque vulnerability.

In the present study we found that only 26.7% of the patients receiving PCI were defined as NGT according to the results of a 75 g OGTT performed for all the patients except known DM patients, and remaining patients had either DM or IGT. Kuhl et al have reported the percentage of NGT was 28% among 1062 ACS patients [31]. Lenzen et al confirmed 24% of NGT patients among 3940 CAD patients [32]. Thus, there have been many patients with potentially unidentified and untreated DM or IGT in this population [31, 32]. The Study To Prevent Non-Insulin-Dependent Diabetes Mellitus (STOP-NIDDM) suggested that the treatment of IGT patients with an α-glucosidase inhibitor was associated with a significant reduction in the risk of cardiovascular disease [5]. This indicates that an abnormality in glucose metabolism should be corrected as early as possible and, therefore, early diagnosis of IGT or DM is essential. Taken together with the findings of the present study, the importance of 75-g OGTT is enhanced in CAD patients, which will allow the timely diagnosis of IGT or latent DM.

In the present study, we also found that fibrous cap thickness positively correlated with HDL-C. The findings similar to our study have been confirmed in the previous studies [33, 34]. Burke et al, have reported that the number of vulnerable plaques were significantly associated with lower serum HDL-C level [33]. Ozaki et al, have shown that HDL-C level is associated with fibrous cap thickness at the culprit lesion of ACS [34]. Although the precise mechanisms whereby HDL-C thicken the fibrous cap is unknown, one of the possible explanations we speculate is the inhibition of MMP-9 activity by HDL-C [35].

### Study limitations

The present study had several limitations. First, this was a subanalysis from the institutional registry at a single hospital which was not conducted specifically to identify the differences of OCT findings, and it had a small sample size. Therefore, selection bias might be present and it may be difficult to make a definitive conclusion from this sample size. Type II error might also exist although we demonstrated no significant differences in clinical backgrounds between the 3 groups. Second, OCT was performed by the operator’s intention and only 1/3 received OCT during the study period. This may also bias the results. However, we think the influence was not significant because operators did not know the results of 75g OGTT at the time of PCI and OCT was mainly selected by the operators who were familiar with handling an OCT device and analyzing OCT images. Third, the exact measurements of the necrotic core and plaque burden on OCT were not possible because of a relatively shallow axial penetration. However, because the most important morphological determinants of plaque vulnerability were superficial, the region of greatest interest was within the imaging range of the current OCT systems. Forth, the OCT data were analyzed in the same institution not in core lab although those were analyzed by independent observers who did not know the clinical backgrounds of the patients. Fifth, the study only included the patients having already developed CAD requiring PCI, which were not the representative patients simply having IGT, NGT, and DM. From the ethical viewpoint, however, it is impossible to perform OCT in patients who do not have significant CAD because of its invasive nature. Finally, the study was a cross sectional observational study which failed to examine the influence of glucose tolerance abnormality on the sequential changes of plaque morphology.

## Conclusion

The vulnerability of coronary plaques of CAD patients having IGT is more significant than that of those having NGT and similar to that of those having DM. This finding may explain the high risk of cardiovascular events in CAD patients with IGT although further investigations such as a long-term follow up study are necessary.

## Supporting Information

S1 Fig Inter-observer reliability measured by the Pearson coefficient. Ten patients were randomly selected and their maximal lipid arch was separately measured.
(TIF)

S2 Fig Intra-observer reproducibility measured by the Pearson coefficient. The same observers re-measured the maximal lipid arch of the selected 10 patients 2 weeks later.
(TIF)

S1 Table All relevant data are available in this table. (XLSX)

## Abbreviations

ACS: acute coronary syndrome
ADA: American diabetes association
CAD: coronary artery disease
DM: diabetes mellitus
DNA: deoxyribonucleic acid
ECG: electro cardio gram
FPG: fasting plasma glucose
GFR: glomerular filtration rate
HDL-C: high-density lipoprotein-cholesterol
IGT: impaired glucose tolerance
LDL-C: low-density lipoprotein-cholesterol
NGT: normal glucose tolerance
OCT: optical coherence tomography
OGTT: oral glucose tolerance test
PCI: percutaneous coronary intervention
PG: plasma glucose
TC: total cholesterol
TCFA: thin-cap fibroatheroma
WHO: World Health Organization
